# Interstitial photodynamic therapy with the second-generation photosensitizer bacteriochlorin a in a rat model for liver metastases.

**DOI:** 10.1038/bjc.1998.353

**Published:** 1998-06

**Authors:** J. P. Rovers, J. J. Schuitmaker, A. L. Vahrmeijer, J. H. van Dierendonck, O. T. Terpstra

**Affiliations:** Department of Surgery, Leiden University Medical Centre, The Netherlands.

## Abstract

**Images:**


*British Journal of Cancer* (1998) **77**(12), 2098–2103
© 1998 Cancer Research Campaign

### Interstitial photodynamic therapy with the second-generation photosensitizer bacteriochlorin *a* in a rat model for liver metastases

**JP Rovers[1], JJ Schuitmaker[2], AL Vahrmeijer[1], JH van Dierendonck[1] and OT Terpstra[1]**

Departments of [1]Surgery and [2]Ophthalmology, Leiden University Medical Centre, PO Box 9600, 2300 RC Leiden, The Netherlands

**Summary** Bacteriochlorin *a* (BCA) is a second-generation photosensitizer that is effective in tumour destruction upon illumination with light of a wavelength of 760 nm. Tissue penetration by light at this wavelength is greater compared with wavelengths at which commonly used photosensitizers are illuminated, making it possible to treat larger tumours. In a model of experimental liver metastases in rats, we measured lesion sizes after interstitial illumination of tumours at different times after intravenous administration of BCA (10 mg $kg^{-1}$ bodyweight), as well as BCA concentrations in liver and tumour tissue. In both, BCA concentrations showed a rapid decline within the first 4 h, followed by a slow decrease over the next 20 h, suggesting biphasic pharmacokinetics. No selective uptake in tumour tissue was observed. A near-linear relationship was found between lesion sizes and liver and tumour BCA concentrations, suggesting that optimal results with photodynamic therapy (PDT) could be obtained by illumination within a short time interval after administration, when tissue concentrations are highest. No severe liver toxicity was observed as indicated by serum ALAT levels. However, in all tumours evaluated, islands of vital-looking cells were present leading to tumour regrowth within 35 days. In view of the obtained lesion diameters of approximately 13 mm after BCA-PDT and the rapid clearance rate of BCA, the concept of a near-infrared absorbing photosensitizer for PDT of liver tumours is a potential interesting strategy.

**Keywords:** photodynamic therapy; photosensitizer; bacteriochlorin *a*; liver neoplasm; interstitial treatment

Photodynamic therapy (PDT) is becoming an accepted treatment for cancer, in which tumour destruction is based on accumulation of a photosensitizing drug in tumour tissue and subsequent illumination with light of a specific wavelength (Gomer, 1991). The light matches an absorption peak of the drug, which becomes activated at illumination. The activated photosensitizer reacts with available oxygen and causes production of reactive oxygen species, such as singlet oxygen. Singlet oxygen can cause direct damage to many cellular sites, such as plasma membranes, microsomes, mitochondria and nuclei (Henderson and Bellnier, 1989). PDT also induces vasoconstriction and blood flow stasis so that tumour cells die of anoxia (Fingar et al, 1990). The combination of a photosensitizer, light and oxygen is required to induce the photochemical reaction.

To date, the most commonly used photosensitizers in clinical studies are haematoporphyrin derivative (HpD), a mixture of various porphyrins and a purified form, Photofrin II. Photofrin is excited with light at a wavelength around 630 nm. Although Photofrin has proven to be an effective photosensitizer, there are some limitations: at a wavelength of 630 nm, tissue penetration of light is limited and light absorption of the photosensitizer is low (Wilson and Patterson, 1986), and Photofrin induces photosensitization of the skin, which makes it necessary for patients to avoid exposure to bright light for 4–8 weeks after treatment (Bellnier and Dougherty, 1989). These limitations can be overcome by 'second-generation' photosensitizers, which usually have a major absorption peak above 650 nm and induce less photosensitization of the skin.

Bacteriochlorin *a* (BCA) is a second-generation photosensitizer and is a very potent photosensitizer. In vitro it photo-oxidizes a number of amino acids and effectively kills mouse fibroblasts and human bladder carcinoma cells (Beems et al, 1987). In vivo, it induces tumour necrosis in hamster Greene melanoma in the anterior eye chamber of white rabbit, in mammary tumours in rat and in RIF tumours in mice (Schuitmaker et al, 1990; van Leengoed et al, 1993; van Geel et al, 1995). BCA has an absorption peak at 760 nm, a wavelength at which tissue penetration is optimal. It is rapidly cleared from tissues and induces only minor skin photosensitivity (van Leengoed et al, 1993).

In the past years, mainly malignancies of the urinary tract, skin, upper respiratory tract and gastrointestinal tract (Fisher et al, 1995) have been treated with PDT, but it has rarely been applied to deep-seated tumours, such as liver tumours. The use of PDT for liver tumours has been restricted as liver tissue accumulates photosensitizers more efficiently than malignant tissue (Bugelski et al, 1981). Superficial illumination will thus result in substantial liver necrosis and, because of limited light penetration, it will be impossible to treat deep-seated or larger solid tumours. This can be overcome by delivering light directly into the tumour – a treatment called interstitial therapy (Marijnissen et al, 1992). The ability of interstitial photodynamic therapy (IPDT) to cause destruction of tumours within the liver has been demonstrated in animal studies, using HpD and Photofrin (Holt et al, 1985; van Hillegersberg et al, 1992). Although tumour destruction was accomplished, there was also considerable damage to surrounding liver tissue, and the amount of tissue that could be treated was limited. New, second-generation photosensitizers could possibly establish a more selective accumulation in tumour tissue, and illumination with light

*Received 14 October 1997*
*Revised 15 January 1998*
*Accepted 17 January 1998*

*Correspondence to:* OT Terpstra

**Table 1** Concentrations of BCA and liver to tumour ratios at different time intervals after intravenous injection

| Time (h) | [Liver tissue] μg mg$^{-1}$ | [Tumour tissue] μg mg$^{-1}$ | Liver/tumour ratio |
|---|---|---|---|
| 1 | 31.90 ± 6.2 | 17.55 ± 2.19 | 2.0 ± 0.3 |
| 2 | 18.97 ± 0.78 | 9.6 ± 0.23 | 2.0 ± 0.2 |
| 4 | 13.78 ± 0.39 | 9.0 ± 0.56 | 1.2 ± 0.1[a] |
| 24 | 5.55 ± 0.27 | 5.78 ± 0.27 | 0.9 ± 0.1[a] |

[Liver tissue], mean ± s.e.m. concentration of BCA in liver tissue; [tumour tissue], mean ± s.e.m. concentration of BCA in tumour tissue. The concentration is expressed as μg mg$^{-1}$ wet tissue. Liver/tumour ratio is expressed as mean ± s.e.m. BCA concentration ratio between liver and tumour tissue for each time point, after determination of the L/T ratio for each animal. [a]Significantly ($P < 0.05$) lower than 1 and 2 h after injection.

**Table 2** Lesion size and percentage of tumour necrosis 2 days after IPDT with BCA

| Time (h) | Lesion size (mm$^2$) | Tumour necrosis (%) |
|---|---|---|
| 1 | 141.8 ± 21.9 | 95.5 ± 4.3 |
| 4 | 95.3 ± 20.7 | 97.8 ± 1.3 |
| 24 | 66.2 ± 26.8 | 72.1 ± 8.5 |
| Control | | 63.0 ± 2.5 |

Lesion size, mean ± s.e.m. of the total amount of damage (liver and tumour) measured at the surface of the liver as calculated with the formula $1/4\ \pi\ R_1\ R_2$. The percentage of tumour necrosis is expressed as mean ± s.e.m. of the percentage of tumour necrosis 2 days after treatment, assessed on microscopic sections of tumours. Animals in the control group were treated either with light or with BCA only.

further penetrating into tissue could result in larger volumes of necrosis. We choose BCA as photosensitizer because of the potential deep penetration of light of 760 nm into tissue. The aim of this study was to investigate the macroscopic and microscopic effect of interstitial illumination on liver and tumour tissue and to correlate induced changes with BCA tissue concentrations at different time intervals after illumination.

### MATERIALS AND METHODS

### Tumour model

Male Wag/Rij rats (Harlan CPB, Zeist, The Netherlands) weighing 245–315 g were used in all experiments. The animals had access to food and water ad libitum. A total of 60 animals was used. In these experiments, we used the CC531 colon adenocarcinoma cell line, which is moderately differentiated, syngeneic and transplantable to Wag/Rij rats. Tumour cells were cultured on RPMI 1640 (Dutch modification) supplemented with 2 mM L-glutamine (Gibco, Grand Island, NY, USA), 10% heat-inactivated fetal calf serum, 100 U ml$^{-1}$ penicillin and 0.1 mg ml$^{-1}$ streptomycin sulphate. At laparotomy, under ether anaesthesia, $5 \times 10^5$ tumour cells were injected subcapsulary into the liver. To determine tissue concentrations, three tumours per rat were induced (left lateral lobe, upper right lobe and lower right lobe), and for the IPDT study one tumour per rat was induced (left lateral lobe). Two weeks after injection, when tumours had reached a diameter of 5–7 mm, they were treated. The experiments were approved by the Animal Welfare Committee of the Leiden University Medical Centre, and animals received care in accordance with established guidelines.

### Photosensitizer

BCA was obtained as described by Schuitmaker et al (1990). Briefly, bacteriochlorophyll *a*, extracted from the photosynthetic bacterium *Rhodospirillum rubrum*, was purified according to the method of Omata and Murata (1983). Saponifying bacteriochlorophyll *a*, as described by Oster et al (1964), yielded bacteriochlorophyllin *a*, which was subsequently subjected to acid hydrolysis. The BCA formed was extracted with ethylacetate. Ethylacetate was evaporated and BCA was lyophilized overnight and stored at –20°C in the dark under nitrogen. Purity of the pigments was checked with thin-layer chromatography and spectrophotometric methods.

BCA was dissolved in a 10% chremophor suspension in 0.9% sterile saline and was injected intravenously in a femoral vein. All animals received a dose of 10 mg kg$^{-1}$ and were kept in subdued light to avoid possible side-effects.

### Determination of photosensitizer concentration

To determine BCA tissue concentrations and to assess concentration ratios between liver and tumour tissue, we used 24 rats, having three tumours in the liver (14 days after inoculation). At 1, 2, 4 and 24 h after BCA administration, the animals were killed and the liver was removed, tumours were dissected and immediately frozen in liquid nitrogen. Liver tissue, from at least 0.5 cm away from the tumour, was also frozen in liquid nitrogen. The samples were stored at –20°C until analysis. Concentrations of BCA in tissue samples were determined using fluorescence measurements. Tissue samples were weighed and homogenized, on ice and under subdued light, in 3 ml of methanol (Baker Chemicals, The Netherlands). The homogenate was centrifuged (2000 r.p.m. for 5 min), and fluorescence in the supernatant was measured with a spectrofluorometer (Aminco SPF 500) at 685 nm (± 10 nm) using an excitation wavelength of 410 nm (± 5 nm). Fluorescence of known BCA concentrations in methanol was measured and plotted in a concentration scale, which was used to determine tissue concentrations. After correction for the weight of the samples, the concentration of BCA was expressed as μg mg$^{-1}$. From each rat, two tumour and two liver measurements were obtained and the concentration ratio between liver and tumour tissue was calculated. The mean concentration and mean concentration ratio ± standard error of the mean (s.e.m.) were calculated for each time point ($n = 5$).

### Interstitial photodynamic therapy

To assess the effect of interstitial illumination, 36 rats bearing one liver tumour were used. They were randomly assigned to four

© Cancer Research Campaign 1998

**Figure 1** Relationship between BCA concentrations and PDT-induced lesions. Lesion size represents the mean lesion sizes ($mm^2$), measured 48 h after PDT treatment, consisting of both liver and tumour damage. BCA concentration represents the mean ± s.e.m. BCA concentration ($\mu g\ mg^{-1}$) in both liver and tumour tissue. Each point represents the mean of five animals. +, Tumour; ▲, liver groups: three experimental groups [BCA + illumination at 1 ($n$ = 6), 4 ($n$ = 12) and 24 ($n$ = 6) h after i.v. injection] and one control group [illumination only ($n$ = 6), BCA injection only ($n$ = 2) and no treatment ($n$ = 4)]. At laparotomy under ether anaesthesia, the liver was exposed and a 17-gauge Venflon (BOC Ohmeda, Sweden) was inserted in the tumour, through which a 600-µm optical fibre with a plain cut fibre tip was positioned. Tumours were illuminated with 760 nm delivered from a custom-made diode laser (Philips Optoelectronic Centre, Eindhoven, The Netherlands), set at a power density of 100 mW $cm^{-2}$. The total amount of energy delivered to each tumour was 100 J (100 mW for 1000 s).

All animals were killed 48 h after treatment, except for six animals treated 4 h after BCA administration and two animals from the control group: these animals were allowed to survive for 35 days to determine the extent of tumour regrowth after treatment. In these remaining rats ($n$ = 6 + 2), a laparotomy was performed 7 days after illumination to measure tumour size and to determine the short-term effect on tumour growth. In all animals, the size of PDT-induced lesions was measured using sliding callipers and calculated with the formula: $1/4\ \pi\ R_1\ R_2$, where $R_1$ and $R_2$ are diameters perpendicular to each other. After fixation in formalin, livers were cut through the largest diameter of the lesion, embedded in paraffin wax, sectioned (2 µm) and stained with haematoxylin and eosin (HE). The percentage of tumour necrosis was assessed at light microscopy by counting 'viable' and 'necrotic cells' in the tumour: using a standard microscopic grid, on one HE-stained section (magnification 100×), all cells at a crossing of lines were evaluated and assessed as being 'viable' or 'necrotic' according to pathologists' guidelines.

**Figure 2** A histological section 2 days after PDT treatment of (**A**) necrotic liver tissue with a zone of viable hepatocytes around the portal vessels (P), whereas the hepatocytes around the central vein (C) did not survive the treatment. (**B**) Evident is the adherence of granulocytes to the vessel wall of a larger vessel in an area of necrotic liver tissue. Magnification 100 ×

Blood samples (0.5 ml) were taken by orbital puncture immediately before and 2 days after treatment to determine levels of serum alkaline phosphatase (AP), aspartate aminotransferase (ASAT) and alanine aminotransferase (ALAT).

**Table 3** Changes in serum levels of alkaline phosphatase, ASAT and ALAT, measured directly before treatment and 2 days after treatment

| Group | AP | | ASAT | | ALAT | |
|---|---|---|---|---|---|---|
| | Before → after | *P*-value | Before → after | *P*-value | Before → after | *P*-value |
| 1 | 74 → 37 | 0.019[a] | 63 → 164 | 0.011[a] | 18 → 82 | 0.000[a] |
| 2 | 121 → 91 | 0.01[a] | 126 → 127 | 0.935 | 31 → 79 | 0.000[a] |
| 3 | 122 → 93 | 0.01[a] | 128 → 67 | 0.01[a] | 32 → 37 | 0.251 |
| Controls | 115 → 93 | 0.014[a] | 86 → 91 | 0.763 | 31 → 36 | 0.53 |

Changes in serum enzyme levels after illumination at different time intervals after BCA administration: group 1 ($n$ = 6), 1 h after BCA administration; 2 ($n$ = 6), 4 h after BCA administration; 3 ($n$ = 6), 24 h after BCA administration. AP, alkaline phosphatase; ASAT, aspartate aminotransferase; ALAT, alanine aminotransferase. [a]Significant difference ($P$ < 0.05).

© Cancer Research Campaign 1998

### Statistical analysis

Tissue concentrations were determined from two measurements and the mean concentration was calculated for each treatment group. Liver (L) to tumour (T) concentration ratios were calculated for each animal and the mean L/T ratio was determined for each treatment group. Values were expressed as mean ± s.e.m. for each time point ($n$ = 6). An analysis of variance (ANOVA) using the least significant difference (LSD) test was used to analyse the relation between time after injection and liver to tumour ratio. A $P$-value of < 0.05 was considered as significant.

### RESULTS

### Tissue concentrations

Concentrations of BCA in liver and tumour tissue are shown in Table 1: 1 h after BCA administration, both liver and tumour tissues contained high concentrations of BCA. The relatively rapid decrease in the first hours after administration was followed by a slow decline, suggesting biphasic pharmacokinetics. Whereas during the first 2 h liver BCA concentrations were twice that of concentrations in tumour tissue, this difference gradually disappeared. L/T ratios at 1 and 2 h were significantly higher ($P < 0.05$) than those at 4 and 24 h after injection, at which time most of the photosensitizer was cleared from liver and tumour tissue.

### Interstitial photodynamic therapy effect

The mean tumour size before treatment was 30.9 ± 2.8 mm$^2$ ($n$ = 36), and there was no significant difference in tumour size between the different treatment groups. Interstitial illumination was well tolerated, and during illumination the liver surface whitened, unless illumination occurred 24 h after BCA administration; this pale aspect changed into dark red immediately after illumination. Two days after treatment, at resection, induced lesions were circular shaped and had a homogeneous white aspect, except for lesions in animals treated 24 h after BCA administration, which were inhomogeneous and irregular. Interstitial illumination of BCA-containing tissue resulted in extensive necrosis visible on the liver surface. Tumours lay within a necrotic area, so lesion sizes represent tumour necrosis as well as necrosis of surrounding liver tissue. Mean lesion sizes are shown in Table 2. Illumination 1 h after BCA administration induced the largest lesions. The mean lesion diameter was 13.5 ± 1.7 mm, with a maximum diameter of 16.2 mm in this group. In the group treated 4 h after BCA administration, lesion sizes were significantly lower than at 1 h. The mean diameter in this group was 11.0 ± 1.3 mm. Lesion sizes in animals treated 24 h after injection were significantly less than at 4 h.

Correlating lesion sizes with tissue concentrations showed a near-linear relation for both liver and tumour tissue (Figure 1).

On microscopic examination, a sharp boundary between necrotic and viable liver tissue was observed, except when illumination was performed 24 h after BCA administration, when necrotic areas were inhomogeneously spread throughout the lesion. Vital-looking hepatocytes were observed around portal veins at the rim of lesions, whereas no such cells were found around central veins (Figure 2). This phenomenon also occurred in animals treated 1 and 4 h after BCA administration, although to a lesser extent; the zone of viable hepatocytes around vessels clearly increased with the time interval after BCA injection. Larger vessels in the liver survived treatment when they were located at the periphery of the lesion, and in all cases, throughout the entire lesions, massive inflammatory responses were seen, as illustrated by adherence of granulocytes to vessel walls (Figure 2).

At the higher BCA concentration levels, more than 95% of tumour tissue had a necrotic aspect assessed 2 days after interstitial illumination, but islands of vital-looking tumour cells could always be identified microscopically. In all treated animals that were allowed to survive beyond 48 h, tumour regrowth occurred. At laparotomy, 7 days after treatment, the mean tumour size in treated animals ($n$ = 6) was 90.1 mm$^2$, and in untreated animals ($n$ = 2) it was 154.3 mm$^2$. However, 35 days after treatment mean tumour sizes were 204.6 and 204.8 mm$^2$ respectively.

Changes of serum AP, ASAT and ALAT levels are shown in Table 3. Illumination 1 h after BCA administration resulted in a significant increase in both ASAT and ALAT levels, with ΔASAT/ΔALAT ratio > 1. Upon illumination 4 hours after administration, there was a significant increase in the ALAT level only (ΔASAT/ΔALAT ratio < 1). After illumination 24 h after BCA administration, no increase in ASAT and ALAT levels occurred.

### DISCUSSION

PDT has proven to be effective in experimental tumour destruction and growth reduction within the liver, using hematoporphyrin derivative (Holt et al, 1985), Photofrin (van Hillegersberg et al, 1992), pheophorbide a (Nishiwaki et al, 1989) and aminolaevulinic acid (Svanberg et al, 1996). Nevertheless, PDT has rarely been used clinically in treatment of liver tumours, mainly because of effective accumulation of photosensitizers in liver tissue. Distribution studies show that photosensitizers are accumulated in high amounts in reticuloendothelial tissue, such as liver, spleen and kidney (Bellnier et al, 1989), and in particular, liver tissue contains high photosensitizer levels after administration (Bown et al, 1986).

Our study showed that BCA concentrations in liver and tumour tissue were highest 1 h after intravenous administration, suggesting that in our model system the optimal time interval for BCA-IPDT treatment would be somewhere within the first 24 h after BCA administration. This could be of clinical relevance, because it implies that photosensitizer injection and interstitial illumination could take place in a single treatment session.

Distribution of BCA resembled the distribution of most other photosensitizers: high tissue levels in the liver and no tumour selectivity. The occurring biphasic pharmacokinetics has also been described by de Smidt et al (1992) for the photosensitizer bisulphonated tetraphenylporphyrine (TPPS-2A): they attributed the biphasic kinetics of TPPS-2A to an initial association of the photosensitizer with the capillary network, followed by redistribution in the tissue surrounding the vessels. As a consequence, PDT-induced tumour-killing upon illumination shortly after photosensitizer administration is likely to be mainly due to 'shut-down' of tumour vessels and less to a direct killing effect upon tumour cells. However, tumour selectivity is of minor importance as long as enough photosensitizer is present at the time of illumination. Evidently, the selectivity of interstitial treatment relies mostly on light tissue penetration. Although IPDT with BCA may induce a rim of surrounding liver tissue necrosis, we observed only a minor increase in serum ALAT and ASAT levels (Table 3). Induction of a rim of normal tissue damage around a tumour is even preferable in oncological treatment.

Because hepatic tumours are believed to be mainly supplied by blood via the hepatic artery (Ackerman et al, 1969), preferential

© Cancer Research Campaign 1998

uptake in tumour tissue could be established by injecting a photosensitizer directly into the hepatic artery (Nishiwaki et al, 1989; Purkiss et al, 1994). An additional advantage of this local administration could be reduction of systemic effects, such as skin photosensitivity.

Interstitial illumination with BCA as photosensitizer resulted in extensive tumour necrosis and necrosis of surrounding liver tissue. Tumours of CC531 cells transplanted to the liver, by subcapsular inoculation with cultured cells, normally grow with about 60% central necrosis. After PDT, in most cases, tumours were completely surrounded by a zone of necrotic liver tissue. Although lesion sizes, 2 days after illumination, were larger at illumination 1 h rather than 4 h after BCA administration, the percentage of tumour necrosis was similar at these time intervals, on average 97%. Twenty-four hours after BCA administration, the amount of BCA was clearly too low to induce a sufficient phototoxic reaction, which has also been described for bacteriochlorophyll-*a*, a precursor of bacteriochlorin *a* (Henderson et al, 1991).

Induced lesions after illumination 1 h after BCA administration ($141.8 \pm 21.9$ mm$^2$) were large considering the use of a single bare fibre with a spot size of 0.6 mm in diameter. Penetration of light into tissue depends on its optical properties and is mainly determined by scattering and absorption, which are wavelength dependent (Parsa et al, 1989). Arnfield et al (1992) showed that, at a wavelength of 789 nm, absorption and scattering coefficients were lower than at a wavelength of 630 nm, resulting in increased penetration of light. Using BCA and light of 760 nm will thus result in larger lesions compared with Photofrin and illumination with 630 nm, which is an advantage of BCA, especially in highly pigmented tissue, such as the liver. Larger tissue volumes can be treated using multiple fibres and modified fibre tips (Arnfield et al, 1986). Feasibility of a multiple-fibre system for use within the liver has already been demonstrated in humans (Purkiss et al, 1993).

On microscopic examination, islands of viable tumour cells could be identified in most cases. A relationship between surviving cells and blood supply of the tumour could not be established, but is suspected. Korbelik et al (1994) were able to establish a relationship between the direct killing effect of PDT and the proximity of tumour cells to the blood supply. In particular, in the case of bacteriochlorophyll-*a*, a decrease in concentrations away from the blood vessels occurred and the greatest killing effect of PDT was inflicted on cells nearest to blood vessels. Additionally, as phototoxicity of PDT also depends on the presence of enough oxygen, a poor vascularization, and thus a reduction in oxygen, will contribute to less tumour cell killing (Henderson and Fingar, 1989). Therefore, both a reduced photosensitizer concentration and hypoxic conditions could explain the resistance to BCA-PDT treatment. The importance of getting 100% cell kill in liver metastases is clearly illustrated by our finding that, despite the induction of 97.8% necrosis, after 35 days tumour sizes did not seem to differ between treated and untreated animals. Optimizing the treatment protocol could be a possibility to obtain complete tumour remission in this tumour model. Another way to improve response rates could be to combine PDT with other treatment modalities, such as chemotherapy or hyperthermia, which have been shown to be synergetic with PDT (Waldow et al, 1985; Freitas et al, 1990; Bremner et al, 1992). Mitomycin c (MMC) given before illumination has been shown to increase tumour response to PDT with BCA in a RIF tumour (van Geel et al, 1995).

Microscopic examination of induced lesions within 'normal' liver tissue showed a sharp border between vital and necrotic liver tissue. A recurrent phenomenon, however, was the presence of vital-looking hepatocytes around portal vessels, whereas hepatocytes around a central vein did not seem to survive treatment. This finding is counterintuitive, as blood within the liver flows from portal vessels to central veins, and thus highest levels of BCA are to be expected around the portal vessels. A possible explanation has been suggested by van Hillegersberg et al (1992), who proposed that survival of hepatocytes is due to a different hepatocyte environment (perfusion) and liver cell enzyme content in the periportal area, preventing induction of oxidative damage by PDT.

In conclusion, our data suggest that optimal results in IPDT for liver tumours with the photosensitizer BCA will be obtained by illumination shortly after BCA administration, and illustrate that illumination with light of 760 nm is a feasible strategy, potentially allowing the treatment of larger tumour volumes.

### ACKNOWLEDGEMENTS

We would like to thank Dr Van Krieken for his help on the histological data and Rob Keijzer for his help with the preparation of the histological material. We would also like to thank Tinneke van der Nat and her colleagues for their help with the animal experiments.

### REFERENCES

Ackerman NB, Lien WM, Konties and Silverman NA (1969) The blood supply of experimental liver metastases: the distribution of hepatic artery and portal vein blood to 'small' and 'large' tumors. *Surgery* **66**: 1067–1072

Arnfield MR, Gonzalez S, Lea P, Tulip J and McPhee MS (1986) Cylindrical irradiation fibre tip for photodynamic therapy. *Lasers Surg Med* **6**: 150–154

Arnfield MR, Mathew RP, Tulip J and McPhee MS (1992) Analysis of tissue optical coefficients using an approximate equation valid for comparable absorption and scattering. *Phys Med Biol* **37**: 1219–1230

Beems EM, Dubbelman TM, Lugtenburg J, Van Best JA, Smeets MF and Boegheim JP (1987) Photosensitizing properties of bacteriochlorophyllin a and bacteriochlorin a, two derivatives of bacteriochlorophyll a. *Photochem Photobiol* **46**: 639–643

Bellnier DA and Dougherty TJ (1989) The time course of cutaneous porphyrin photosensitization in the murine ear. *Photochem Photobiol* **49**: 369–372

Bellnier DA, Ho YK, Pandey RK, Missert JR and Dougherty TJ (1989) Distribution and elimination of Photofrin II in mice. *Photochem Photobiol* **50**: 221–228

Bown SG, Tralau CJ, Coleridge-Smith PD, Akdemir D and Wieman TJ (1986) Photodynamic therapy with porphyrin and phthalocyanine sensitization: quantitative studies in normal rat liver. *Br J Cancer* **54**: 43–52

Bremner JC, Adams GE, Pearson JK, Sansom JM, Stratford IJ, Bedwell J, Bown SG, Macrobert AJ and Phillips D (1992) Increasing the effect of photodynamic therapy on the RIF-1 murine sarcoma, using the bioreductive drugs RSU1069 and RB6145. *Br J Cancer* **66**: 1070–1076

Bugelski PJ, Potter CW and Dougherty TJ (1981) Autoradiographic distribution of hematoporphyrin derivative in normal and tumor tissue of the mouse. *Cancer Res* **41**: 4606–4612

De Smidt PC, Versluis AJ and Van Berkel TJC (1992). Transport of sulfonated tetraphenylporphine by lipoproteins in the hamster. *Biochem Pharmacol* **43**: 2567–2573

Fingar VH, Wieman TJ and Doak KW (1990) Role of thromboxane and prostacyclin release on photodynamic therapy-induced tumour destruction. *Cancer Res* **50**: 2599–2603

Fisher AMR, Murphree AL and Gomer CJ (1995) Clinical and preclinical photodynamic therapy. *Lasers Surg Med* **17**: 2–31

Freitas I, Pontiggia P, Baronzio GF and McLaren JR (1990) Perspectives for the combined use of photodynamic therapy and hyperthermia in cancer patients. *Adv Exp Med Biol* **267**: 511–520

Gomer CJ (1991) Preclinical examination of first and second generation photosensitizers used in photodynamic therapy (Review). *Photochem Photobiol* **54**: 1093–1107

Henderson BW and Bellnier DA (1989) Tissue localization of photosensitizers and the mechanism of photodynamic tissue destruction. *Ciba Found Symp* **146**: 113–130

 *© Cancer Research Campaign 1998*

Henderson BW and Fingar VH (1989) Oxygen limitation of direct tumour cell kill during photodynamic treatment of a murine tumor model. *Photochem Photobiol* **49**: 299–304

Henderson BW, Sumlin AB, Owczarczak BL and Dougherty TJ (1991) Bacteriochlorophyll-a as photosensitizer for photodynamic treatment of transplantable murine tumours. *J Photochem Photobiol B* **10**: 303–313

Holt S, Tulip J, Hamilton D, Cummins J, Fields A and Dick C (1985) Experimental laser phototherapy of the morris 7777 hepatoma in the rat. *Hepatology* **5**: 175–180

Korbelik M and Krosl G (1994) Cellular levels of photosensitisers in tumours: the role of proximity to the blood supply. *Br J Cancer* **70**: 604–610

Marijnissen JP, Versteeg JA, Star WM and Van Putten WL (1992) Tumor and normal tissue response to interstitial photodynamic therapy of the rat R-1 rhabdomyosarcoma. *Int J Radiat Oncol Biol Phys* **22**: 963–972

Nishiwaki Y, Nakamura S and Sakaguchi S (1989) New method of photosensitizer accumulation for photodynamic therapy in an experimental liver tumour. *Lasers Surg Med* **9**: 254–263

Omata T and Murata N (1983) Preparation of chlorophyll a, chlorophyll b and bacteriochlorophyll a by column chromatography with DEAE Sepharose C1-6B. *Plant Cell Physiol* **24**: 1093–1100

Oster G, Broyde B and Bellin JS (1964) Spectral properties of chlorophyllin a. *J Am Chem Soc* **5**: 1309–1313

Parsa P, Jacques SL and Nishioka NS (1989) Optical properties of rat liver between 350 and 2200 nm. *Appl Opt* **28**: 2325–2330

Purkiss SF, Dean R, Allardice JT, Grahn MF and Williams NS (1993) An interstitial light delivery system for photodynamic therapy within the liver. *Lasers Med Sci* **8**: 253–257

Purkiss SF, Hutton M and Williams NS (1994) A comparison of photosensitizer administration routes for interstitial photodynamic therapy of the liver. *Lasers Med Sci* **9**: 291–296

Schuitmaker JJ, Van Best JA, Van Delft JL, Dubbelman TM, Oosterhuis JA and De Wolff-Rouendaal D (1990) Bacteriochlorin a, a new photosensitizer in photodynamic therapy. In vivo results. *Invest Ophthalmol Vis Sci* **31**: 1444–1450

Svanberg K, Liu DL, Wang I, Andersson-Engels S, Stenram U and Svanberg S (1996) Photodynamic therapy using intravenous d-aminolaevulinic acid-induced protoporphyrin IX sensitisation in experimental hepatic tumours in rats. *Br J Cancer* **74**: 1526–1533

Van Geel IP, Oppelaar H, Oussoren YG, Schuitmaker JJ and Stewart FA (1995) Mechanisms for optimising photodynamic therapy: second-generation photosensitisers in combination with mitomycin C. *Br J Cancer* **72**: 344–350

Van Hillegersberg R, Marijnissen JP, Kort WJ, Zondervan PE, Terpstra OT and Star WM (1992) Interstitial photodynamic therapy in a rat liver metastasis model. *Br J Cancer* **66**: 1005–1014

Van Leengoed HL, Schuitmaker JJ, Van Der Veen N, Dubbelman TM and Star WM (1993) Fluorescence and photodynamic effects of bacteriochlorin a observed in vivo in 'sandwich' observation chambers. *Br J Cancer* **67**: 898–903

Waldow SM, Henderson BW and Dougherty TJ (1985) Potentiation of photodynamic therapy by heat: effect of sequence and time interval between treatments in vivo. *Lasers Surg Med* **5**: 83–94

Wilson BC and Patterson MS (1986) The physics of photodynamic therapy. *Phys Med Biol* **31**: 327–360

© Cancer Research Campaign 1998


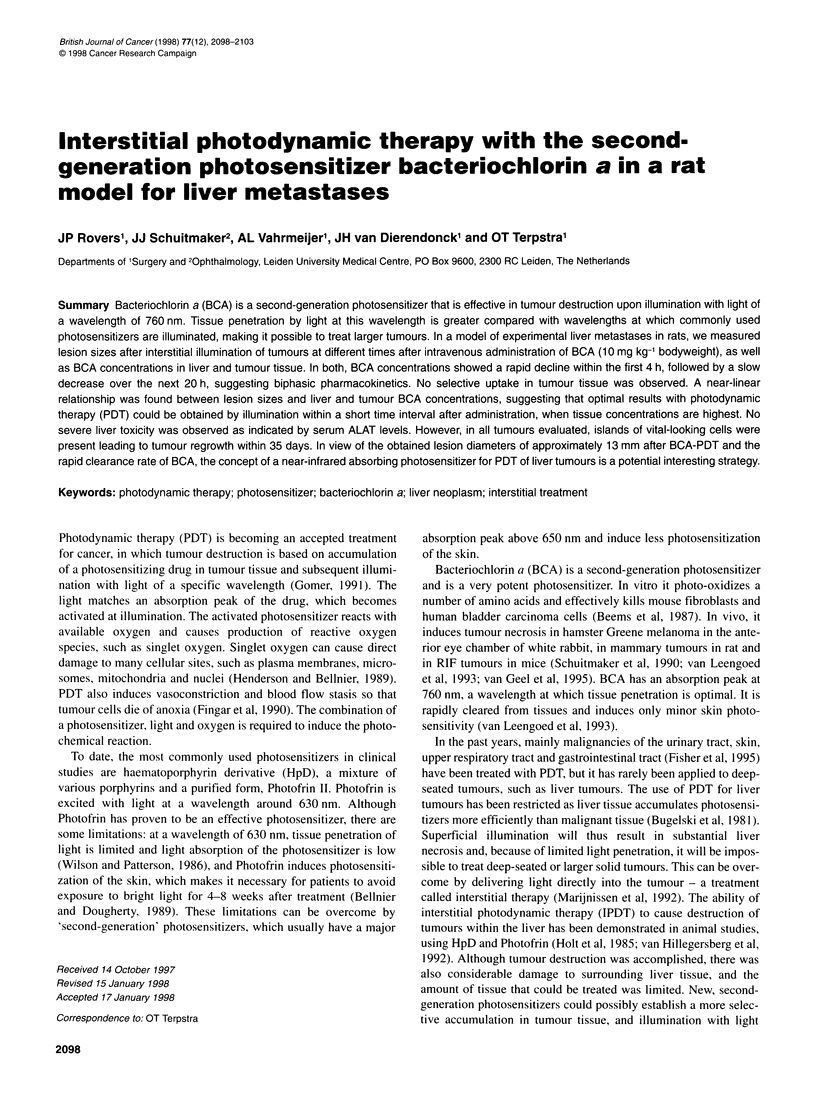

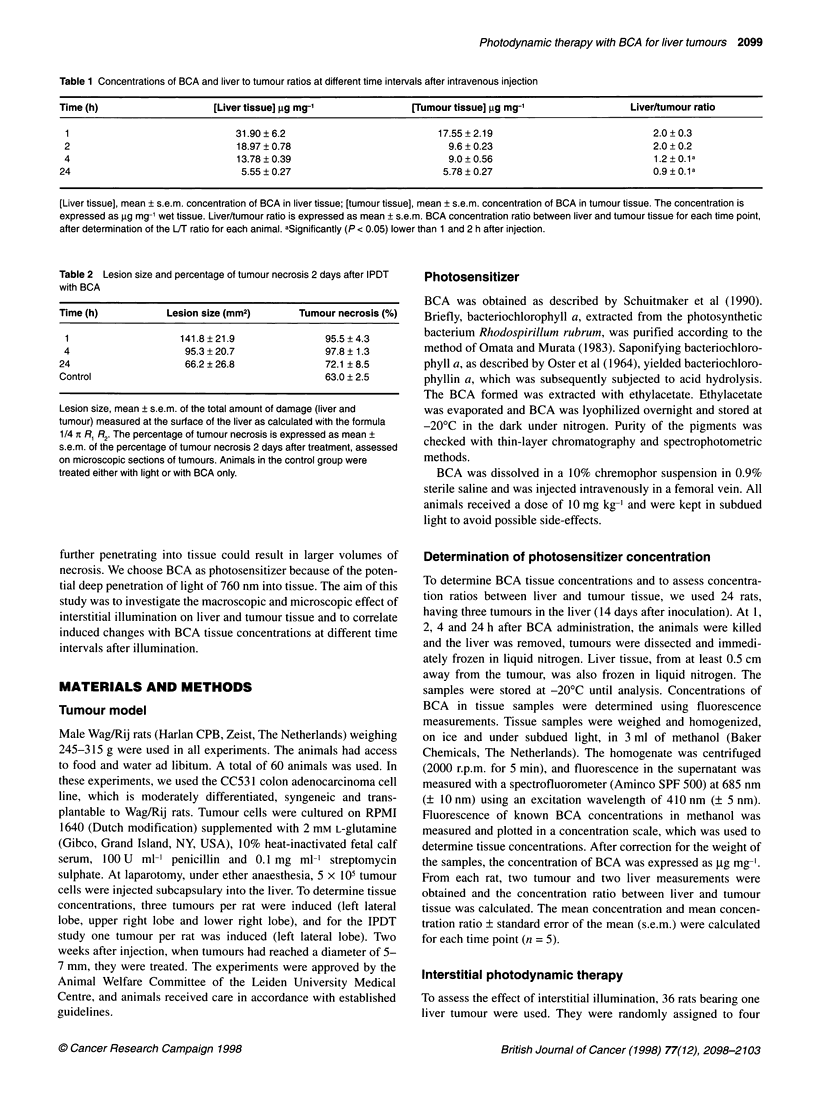

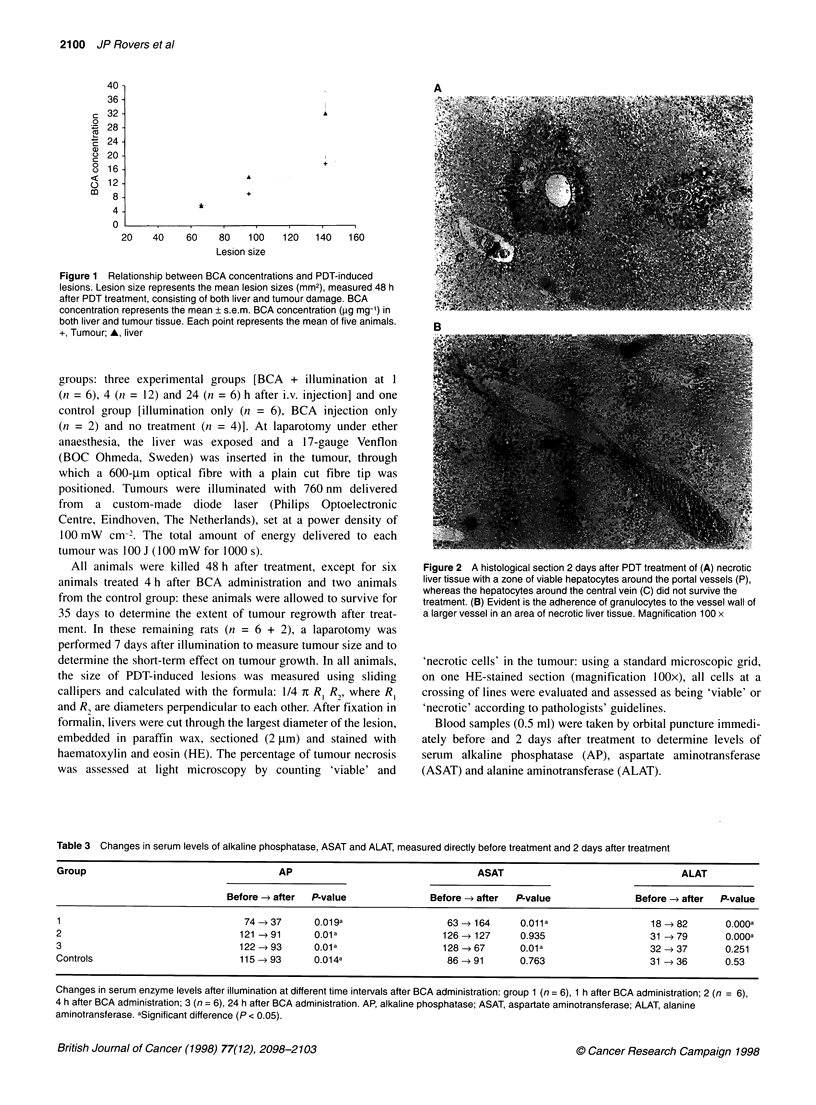

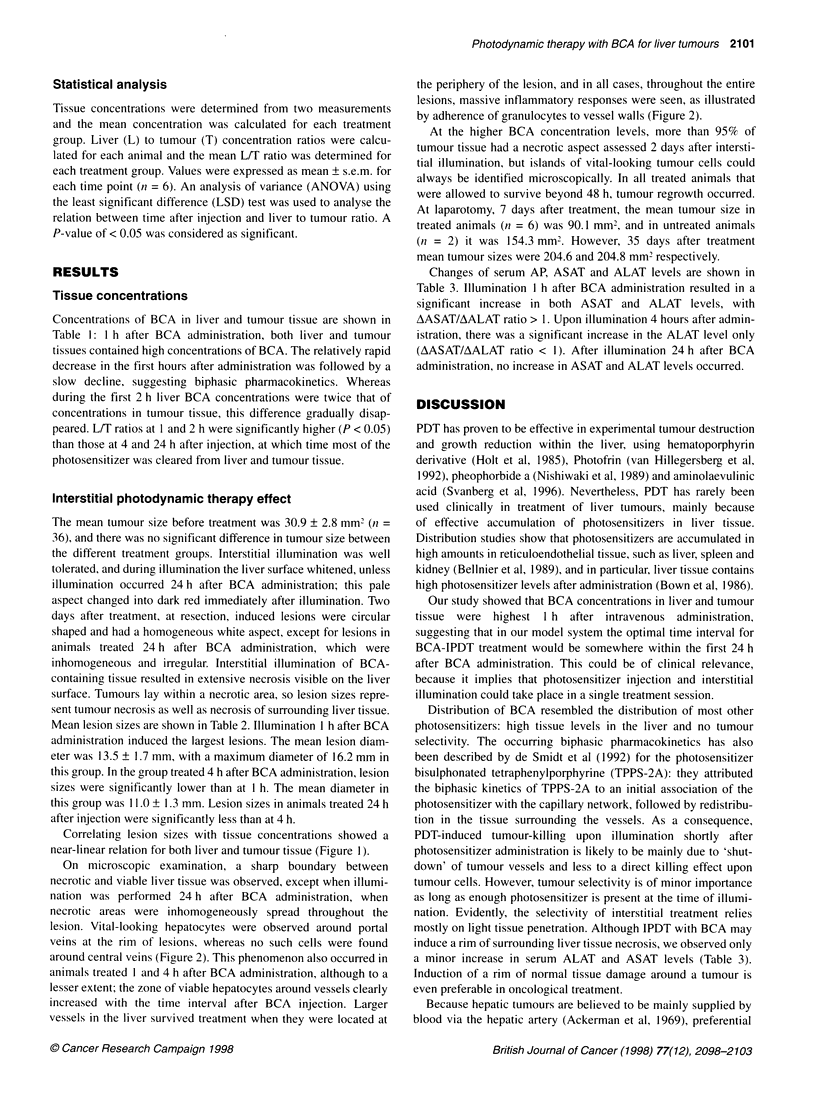

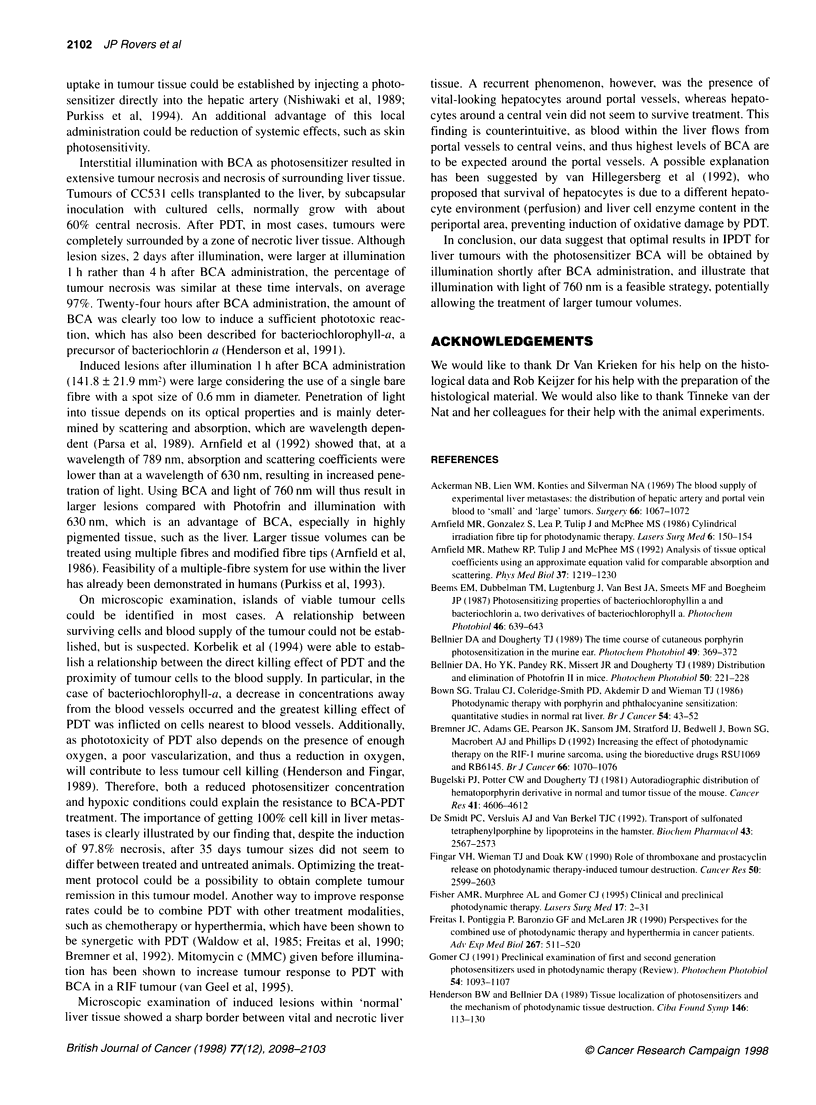

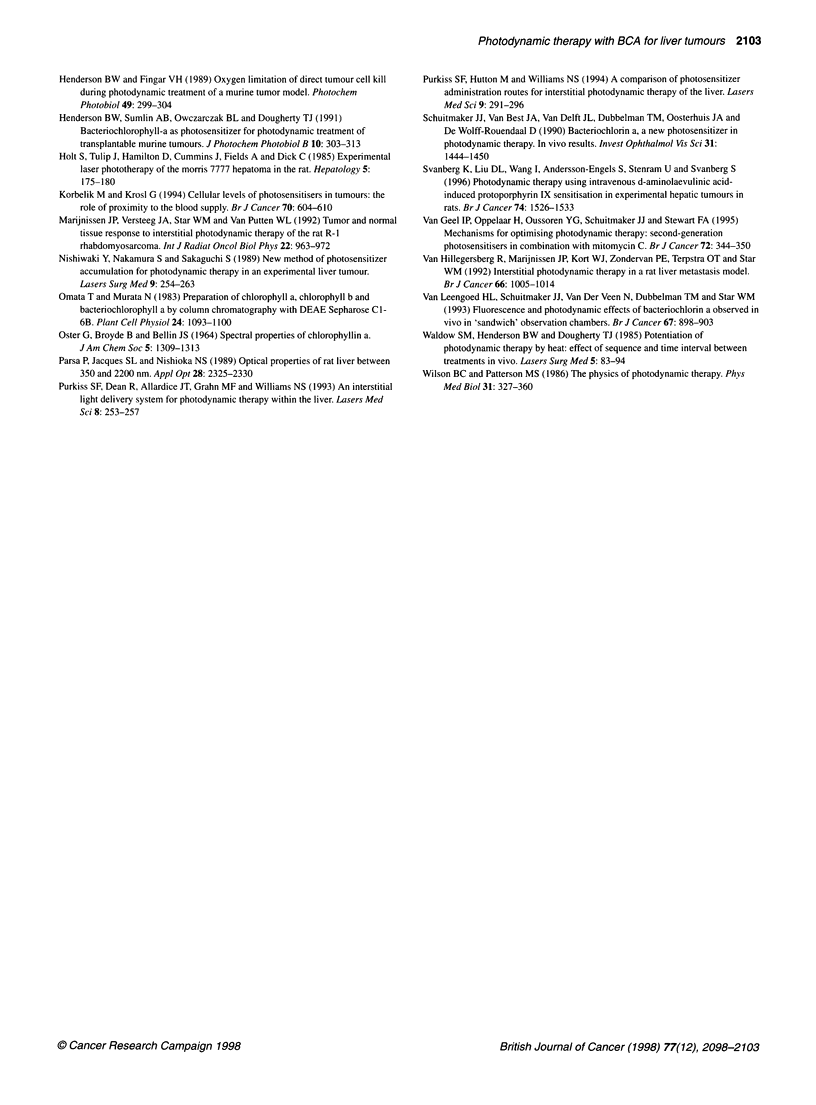


## References

[OCR_00569] Ackerman N. B., Lien W. M., Kondi E. S., Silverman N. A. (1969). The blood supply of experimental liver metastases. I. The distribution of hepatic artery and portal vein blood to "small" and "large" tumors.. Surgery.

[OCR_00578] Arnfield M. R., Mathew R. P., Tulip J., McPhee M. S. (1992). Analysis of tissue optical coefficients using an approximate equation valid for comparable absorption and scattering.. Phys Med Biol.

[OCR_00574] Arnfield M., Gonzalez S., Lea P., Tulip J., McPhee M. (1986). Cylindrical irradiator fiber tip for photodynamic therapy.. Lasers Surg Med.

[OCR_00583] Beems E. M., Dubbelman T. M., Lugtenburg J., Van Best J. A., Smeets M. F., Boegheim J. P. (1987). Photosensitizing properties of bacteriochlorophyllin a and bacteriochlorin a, two derivatives of bacteriochlorophyll a.. Photochem Photobiol.

[OCR_00590] Bellnier D. A., Dougherty T. J. (1989). The time course of cutaneous porphyrin photosensitization in the murine ear.. Photochem Photobiol.

[OCR_00594] Bellnier D. A., Ho Y. K., Pandey R. K., Missert J. R., Dougherty T. J. (1989). Distribution and elimination of Photofrin II in mice.. Photochem Photobiol.

[OCR_00597] Bown S. G., Tralau C. J., Smith P. D., Akdemir D., Wieman T. J. (1986). Photodynamic therapy with porphyrin and phthalocyanine sensitisation: quantitative studies in normal rat liver.. Br J Cancer.

[OCR_00602] Bremner J. C., Adams G. E., Pearson J. K., Sansom J. M., Stratford I. J., Bedwell J., Bown S. G., MacRobert A. J., Phillips D. (1992). Increasing the effect of photodynamic therapy on the RIF-1 murine sarcoma, using the bioreductive drugs RSU1069 and RB6145.. Br J Cancer.

[OCR_00609] Bugelski P. J., Porter C. W., Dougherty T. J. (1981). Autoradiographic distribution of hematoporphyrin derivative in normal and tumor tissue of the mouse.. Cancer Res.

[OCR_00619] Fingar V. H., Wieman T. J., Doak K. W. (1990). Role of thromboxane and prostacyclin release on photodynamic therapy-induced tumor destruction.. Cancer Res.

[OCR_00624] Fisher A. M., Murphree A. L., Gomer C. J. (1995). Clinical and preclinical photodynamic therapy.. Lasers Surg Med.

[OCR_00628] Freitas I., Pontiggia P., Baronzio G. F., McLaren J. R. (1990). Perspectives for the combined use of photodynamic therapy and hyperthermia in cancer patient.. Adv Exp Med Biol.

[OCR_00633] Gomer C. J. (1991). Preclinical examination of first and second generation photosensitizers used in photodynamic therapy.. Photochem Photobiol.

[OCR_00647] Henderson B. W., Fingar V. H. (1989). Oxygen limitation of direct tumor cell kill during photodynamic treatment of a murine tumor model.. Photochem Photobiol.

[OCR_00652] Henderson B. W., Sumlin A. B., Owczarczak B. L., Dougherty T. J. (1991). Bacteriochlorophyll-a as photosensitizer for photodynamic treatment of transplantable murine tumors.. J Photochem Photobiol B.

[OCR_00657] Holt S., Tulip J., Hamilton D., Cummins J., Fields A., Dick C. (1985). Experimental laser phototherapy of the Morris 7777 hepatoma in the rat.. Hepatology.

[OCR_00662] Korbelik M., Krosl G. (1994). Cellular levels of photosensitisers in tumours: the role of proximity to the blood supply.. Br J Cancer.

[OCR_00666] Marijnissen J. P., Versteeg J. A., Star W. M., van Putten W. L. (1992). Tumor and normal tissue response to interstitial photodynamic therapy of the rat R-1 rhabdomyosarcoma.. Int J Radiat Oncol Biol Phys.

[OCR_00671] Nishiwaki Y., Nakamura S., Sakaguchi S. (1989). New method of photosensitizer accumulation for photodynamic therapy in an experimental liver tumor.. Lasers Surg Med.

[OCR_00699] Schuitmaker J. J., van Best J. A., van Delft J. L., Dubbelman T. M., Oosterhuis J. A., de Wolff-Rouendaal D. (1990). Bacteriochlorin a, a new photosensitizer in photodynamic therapy. In vivo results.. Invest Ophthalmol Vis Sci.

[OCR_00705] Svanberg K., Liu D. L., Wang I., Andersson-Engels S., Stenram U., Svanberg S. (1996). Photodynamic therapy using intravenous delta-aminolaevulinic acid-induced protoporphyrin IX sensitisation in experimental hepatic tumours in rats.. Br J Cancer.

[OCR_00727] Waldow S. M., Henderson B. W., Dougherty T. J. (1985). Potentiation of photodynamic therapy by heat: effect of sequence and time interval between treatments in vivo.. Lasers Surg Med.

[OCR_00732] Wilson B. C., Patterson M. S. (1986). The physics of photodynamic therapy.. Phys Med Biol.

[OCR_00614] de Smidt P. C., Versluis A. J., van Berkel T. J. (1992). Transport of sulfonated tetraphenylporphine by lipoproteins in the hamster.. Biochem Pharmacol.

[OCR_00712] van Geel I. P., Oppelaar H., Oussoren Y. G., Schuitmaker J. J., Stewart F. A. (1995). Mechanisms for optimising photodynamic therapy: second-generation photosensitisers in combination with mitomycin C.. Br J Cancer.

[OCR_00718] van Hillegersberg R., Marijnissen J. P., Kort W. J., Zondervan P. E., Terpstra O. T., Star W. M. (1992). Interstitial photodynamic therapy in a rat liver metastasis model.. Br J Cancer.

[OCR_00723] van Leengoed H. L., Schuitmaker J. J., van der Veen N., Dubbelman T. M., Star W. M. (1993). Fluorescence and photodynamic effects of bacteriochlorin a observed in vivo in 'sandwich' observation chambers.. Br J Cancer.

